# Prevalence of chronic idiopathic musculoskeletal pain and headache in adolescence: a population based Norwegian study

**DOI:** 10.1186/1546-0096-10-S1-A77

**Published:** 2012-07-13

**Authors:** Gry B Hoftun, Pål R Romundstad, John-Anker Zwart, Marite Rygg

**Affiliations:** 1 Norwegian University of Science and Technology, Faculty of Medicine, Trondheim, Norway; 2 Norwegian University of Science and Technology, Faculty of Medicine/Oslo University Hospital, Ullevål, and University of Oslo, Trondheim/Oslo, Norway; 3 Norwegian University of Science and Technology, Faculty of Medicine/St.Olav’s Hospital, Trondheim, Norway

## Purpose

The aim of this study was to determine the prevalence of chronic idiopathic musculoskeletal pain and headache among adolescents in relation to age and gender.

## Methods

A population-based, cross-sectional study was carried out in Nord-Trøndelag county, Norway, during the years 2006-2008. All adolescents aged 12-19 years were invited to participate in the youth part of the Nord-Trøndelag Health Study [“Helseundersøkelsen i Nord-Trøndelag (HUNT)”]. Response rate was 78% (8200 responders out of 10.485 invited). The adolescents completed a comprehensive questionnaire including questions about whether they had experienced any headaches and/or any musculoskeletal pain during the last three months, not related to any known disease or injury. They were asked to indicate the location of pain (neck/shoulders, upper back, lower back/buttocks, chest, upper and/or lower extremities). The participants were also asked to indicate how often they had experienced pain in these areas (seldom or never, once a month, once a week, more than once a week, almost every day). The final study population, with complete pain questionnaires, consisted of 7373 adolescents aged 13-18 years.

## Results

Pain at least once a week during the last three months was reported by 44% (95% CI 43-45%), including 54% (95% CI 53-56%) of the girls and 34% (95% CI 33-36%) of the boys. The prevalence of headache at least once a week during the last three months was 22% (95% CI 21-23%), including 30% (95% CI 29-31%) of the girls and 14% (95% CI 13-15%) of the boys, and for musculoskeletal pain 33% (95% CI 32-34%), girls 40% (95% CI 38-42%) and boys 27% (95% CI 25-28%). Pain in the neck/shoulder region was the most commonly affected musculoskeletal region, reported by 17% (22% of the girls and 12% of the boys). Pain prevalence was higher in girls than in boys in all age groups and for all pain categories. The prevalence of pain increased with age in most pain categories and locations, except for pain in upper and lower extremities.

## Conclusion

Chronic pain, defined as pain at least once a week present for at least three months, is common among Norwegian adolescents, with higher prevalence in girls than boys. Musculoskeletal pain shows the highest prevalence, and is reported by one third of the study population.

## Disclosure

Gry B Hoftun: None; Pål R Romundstad: None; John-Anker Zwart: None; Marite Rygg: None.

